# Optimizing Bay Scallop (*Argopecten irradians*) Product Quality: Moderate Freezing as an Effective Strategy for Improving Adductor Muscle Gel Properties

**DOI:** 10.3390/foods14081371

**Published:** 2025-04-16

**Authors:** Kexin Chang, Yufan Lin, Sijia Huang, Xinru Fan, Yongsheng Ma, Meng Li, Qiancheng Zhao

**Affiliations:** 1 College of Food Science and Engineering, Dalian Ocean University, Dalian 116023, China; kk518815@163.com (K.C.); l17304516097@163.com (Y.L.); 15898207377@163.com (S.H.); fanxinru@dlou.edu.cn (X.F.); mayo@dlou.edu.cn (Y.M.); 2 Liaoning Provincial Marine Healthy Food Engineering Research Centre, Dalian 116023, China; 3 Dalian Key Laboratory of Marine Bioactive Substance Development and High Value Utilization, Dalian 116023, China

**Keywords:** short-term frozen storage, heat-induced gel, gelling properties, myofibrillar protein, bay scallop

## Abstract

The bay scallop (*Argopecten irradians*) adductor is an attractive raw material for the production of surimi-like products. The gelling properties of raw materials directly affect the quality of surimi-like products. To assess the potential of processing frozen bay scallop adductors into surimi-like products, the effects of short-term freezing treatment on the endogenous transglutaminase (TGase) activity, myofibrillar protein (MP) structure and gelling properties of bay scallop adductors were investigated during 14 days of frozen storage (−18 °C). The results showed that TGase activity in adductor muscles increased significantly during the first 7 days. After 7–14 days, the carbonyl and sulfhydryl contents of the MPs notably changed (increased then decreased). The β-turn content of the MPs increased, indicating stretching and flexibility. Surface hydrophobicity, fluorescence intensity and sodium dodecyl sulfate–polyacrylamide gel electrophoresis (SDS-PAGE) analysis demonstrated changes in the tertiary structure of the MPs. Compared with gels from fresh samples, gels from scallop adductors frozen for 1 day presented significantly better texture characteristics (breaking force, gel strength, hardness, springiness, cohesiveness, chewiness) and higher water-holding capacity (*p* < 0.05). However, these properties significantly decreased on the 7th and 14th days (*p* < 0.05). Microstructural analysis revealed a more compact gel network from 1-day-frozen adductor muscles. These changes in TGase activity and MP structure are key factors influencing the gelling properties of frozen bay scallop adductors. This study provides new insights for improving gel properties during the frozen storage of bay scallop adductors.

## 1. Introduction

The scallop is the most important bivalve mollusk in international trade [1]. China leads the world in scallop aquaculture, with more than 1.8 million tons of scallops from aquaculture harvested in 2023 [2]. Bay scallops (*Argopecten irradians*) are widely produced and consumed in China because their growth rate is faster than that of other commercial species, such as *Patinopecten yessoensis* and *Chlamys farreri* [3]. The bay scallop adductor is abundantly available and nutritional, with gel-forming ability, a white mantle, a low market price and a short life cycle, making it an attractive raw material for the production of surimi-like products [4,5,6].

Fresh scallop adductor muscle is extremely perishable because of protein and lipid oxidation, active endogenous enzymes and microbial growth, and it has a refrigerated shelf life of only approximately 2 days [7]. To achieve a longer shelf life of this perishable aquatic material, freezing is the standard practice to maintain quality [8]. Thus, frozen scallop adductors are more conducive to product development than fresh materials. Liu et al. [9] reported that during frozen storage, the quality of scallop adductor muscles significantly deteriorates, resulting in texture alteration and the presence of drip fluid. Moreover, numerous reports have indicated that frozen storage deteriorates the gelling properties of various surimi or minced aquatic material muscles [10,11,12,13]. However, few reports exist on the changes in the gelling properties of frozen bay scallop adductor muscles.

Heat treatment is currently the main method for inducing surimi or minced material muscle gelation. The gel-forming process can be divided into the suwari, modori and kamaboko stages [14]. During the suwari phase, which is the gel-setting stage, surimi transitions from a sol state to a gel with low hardness and high elasticity, primarily occurring within the temperature range of 0–50 °C. During the gel-setting phase, transglutaminase (TGase) facilitates the covalent cross-linking between γ-carboxamide groups and primary amines, thereby promoting the aggregation of myofibrillar protein (MP) to form gels. When heated to 50–70 °C, the gel transitions into the modori stage, during which endogenous protease (such as cysteine and serine proteases) activity peaks, leading to the degradation of MPs and the disruption of the gel network structure. Upon heating to 70–90 °C, the gel reaches the kamaboko stage, characterized by an enhancement in gel strength, which supports the formation of a more ordered three-dimensional gel network structure [15]. The properties of heat-induced gels, such as gel texture, water-holding capacity (WHC) and whiteness, are a very important concern when determining the quality of surimi-like products, influencing consumer experience and the textural characteristics of products [16].

MPs notably affect the gelling properties of fish surimi and bay scallop adductor muscles [17]. However, the heat-induced gel of scallop adductor muscles contains not only salt-soluble proteins, which are mainly composed of MPs, but also water-soluble proteins [18]. Endogenous proteases in water-soluble proteins, such as endogenous TGase, can also affect gel properties. The extent of gelation catalyzed by TGase is also affected by MP structure and conformation [19]. During frozen storage, freezing-induced MP denaturation and oxidation play crucial roles in the degradation of gel quality [20]. In addition, endogenous TGase activity significantly decreases during long-term frozen storage [21]. However, the specific effects of short-term frozen storage (≤14 days) on TGase activity and MP structure, which contribute to the gelling properties of the bay scallop adductor, remain unclear.

Therefore, this study investigated the changes in TGase activity and alterations in the oxidation and structure of MPs in bay scallop adductor muscles during short-term frozen storage (0, 1, 7 and 14 d). Additionally, the study monitored the fluctuations in the texture characteristics, WHC, whiteness and microstructure of heat-induced gels from bay scallop adductor muscles during frozen storage. The associations among TGase activity, MP oxidation, MP structure and gelling properties were also assessed. This study potentially provides a theoretical foundation for the optimization of surimi-like scallop production.

## 2. Materials and Methods

### 2.1. Materials and Sample Collection

Live bay scallop (*Argopecten irradians*) samples of approximately 40 kg were purchased from a local retail market in Dalian, Liaoning Province, China. The average mass of the single scallops used for the experiment was 100 ± 50 g, and the longest axis diameter was approximately 10 ± 2 mm. Bay scallops were transported to the laboratory in a foam box with ice within 20 min. The fresh adductor was manually removed from the shells and cleaned once with cold pure water to remove impurities. After draining, some fresh adductors (approximately 1.5 kg) were immediately subjected to various physicochemical analyses, whereas some samples were separated randomly into three batches of 1.5 kg each and individually stored in polyethylene bags at −18 °C for 1, 7 and 14 d before analysis.

### 2.2. Transglutaminase (TGase) Activity of Bay Scallop Adductor

TGase activity was assayed via the method of Yongsawatdigul et al., with slight modifications [22]. Fresh and thawed bay scallop adductor samples (5 g) were homogenized in 3 volumes of extraction buffer (10 mM NaCl, 5 mM EDTA, 2 mM dithiothreitol (DTT) and 10 mM Tris–HCl, pH 7.5). The homogenates were centrifuged at 16,000× *g* (Hunan Xiangyi Laboratory Instrument Development Co., Changsha, China) at 4 °C for 30 min. The supernatant was used as the crude enzyme extract. The assay mixture contained 1.0 mg/mL N-N’-demethylated casein (DMC), 15 μM monodansylcadaverine (MDC), 5 mM CaCl_2_, 3 mM DTT, 50 mM Tris–HCl (pH 7.5) and 100 μL crude enzyme. After incubation at 37 °C for 10 min, the EDTA solution was added to a final concentration of 20 mM to stop the reaction. The fluorescence intensity was measured with excitation and emission wavelengths of 350 and 380 nm, respectively, via an F-2700 fluorescence spectrophotometer (Hitachi High-Tech Science Corporation, Tokyo, Japan). The enhancing factor, indicating the degree of fluorescence enhancement of the dansyl group after incorporation into DMC, was determined in our study to be 1.78. One unit of TGase activity was defined as the amount of enzyme that catalyzed the incorporation of 1 nmol of MDC into DMC per min.

### 2.3. Preparation of MPs from Bay Scallop Adductor

The MPs were extracted from scallop adductor muscles according to the methods of Yi et al. [23]. The minced adductor muscle (approximately 2 g) was homogenized at 4000 rpm for 2 min with 4 volumes of buffer (10 mM Tris, pH 7.2). The mixture was subsequently centrifuged at 5000× *g* for 10 min at 4 °C, after which the supernatant was removed. The above operation was repeated 3–4 times. The obtained sediment was resuspended in buffer (0.6 M NaCl, 20 mM Tris–HCl, pH 7.0) and then homogenized. The homogenate was incubated for 1 h at 4 °C and then centrifuged for 5 min. Then, the supernatant that contained myofibrillar protein was collected and stored at 4 °C for analysis within one day.

### 2.4. Carbonyl Content

The carbonyl content was measured according to methods of Xia et al., with some modifications [24]. The protein concentration was adjusted to 2 mg/mL, and 10 mM 2,4-dinitrophenylhydrazine (DNPH) dissolved in 2 M NaCl was added. The mixture was placed in the dark at 37 °C for 1 h with vortexing every 15 min, mixed with 20% trichloroacetic acid (TCA) and centrifuged at 8000× *g* for 10 min at 4 °C. The precipitate was washed with ethyl acetate/ethanol (1:1 *v*/*v*) until colorless and dissolved in 6 M guanidine containing 20 mM phosphate buffer (pH 6.5). The samples were centrifuged again with the same parameters, and the absorbance was measured at 370 nm. The carbonyl content is expressed as nanomoles of carbonyl per milligram of protein using an absorption coefficient of 22,000 M^−1^ cm^−1^ for the protein hydrazones.

### 2.5. Sulfhydryl Content

The sulfhydryl contents of the scallop adductor MP samples were determined as described by Pan et al. [25]. The MP solutions (4 mg/mL) were mixed with 9 volumes of Tris–HCl containing 8 M carbamide, 2 g SDS per liter and 10 mM EDTA (pH 6.8). Then, 4 mL of the mixture was mixed with 0.4 mL of 10 mM 5,5′-dithiobis (DTNB). The mixture was incubated at 4 °C for 25 min, and the absorbance was detected at 412 nm. The sulfhydryl content is expressed as nanomoles of sulfhydryl per milligram of protein by using the molar extinction coefficient (13,600 M^−1^ cm^−1^).

### 2.6. Fourier-Transform Infrared (FTIR) Spectroscopy

The FTIR spectra of the MPs were determined via a NEXUS670 FTIR spectrometer (PerkinElmer, Hopkinton, MA, USA) according to the methods of Hu et al. [26]. Pure potassium bromide (KBr) was dried to a constant weight and scanned as a background. Free-dried MP (2 mg) was mixed with KBr (150 mg) and then pressed into slices for FTIR measurements. The scanning range was 4000 to 400 with a resolution of 4 cm^−1^. To further evaluate the changes in amide Ι, Peak Fit version 4.0 software was used to analyze the secondary structure of the MPs, and a Gaussian peak-fitting algorithm was used. The secondary structure content was calculated from the integral area.

### 2.7. Surface Hydrophobicity

Protein surface hydrophobicity was assayed via the use of 8-anilino-1-naphthalenesulfonic acid (ANS) as the fluorescence probe according to the methods of You et al., with modifications [27]. The myofibrillar protein solution was diluted to 0.2, 0.3, 0.5 and 1.0 mg/mL with 0.6 M NaCl–10 mM phosphate buffer (pH 6.0). Then, 20 μL of 8 mM ANS was added to the diluted protein mixture for a 10 min reaction in the dark. The fluorescence intensities (FIs) of the ANS conjugates were measured immediately with an F-2700 fluorescence spectrophotometer (Hitachi High-Tech Science Corporation, Tokyo, Japan) at an excitation wavelength of 390 nm and an emission wavelength of 420 nm. The initial slope of the plot of FI vs. the concentration of myofibrillar protein solution was used as an index of protein surface hydrophobicity.

### 2.8. Fluorescence Intensity

The fluorescence intensities of the MP samples were assayed according to a previously described method [9] via an F-2700 fluorescence spectrophotometer (Hitachi High-Tech Science Corporation, Tokyo, Japan). In brief, the MP solution was diluted to 0.4 mg/mL in 15 mM phosphate-buffered saline buffer (pH = 6.25) containing 0.6 M NaCl. The fluorescence data were collected in the wavelength range of 300–400 nm, and the excitation wavelength was set at 283 nm at room temperature.

### 2.9. Sodium Dodecyl Sulfate–Polyacrylamide Gel Electrophoresis

The MP profiles were analyzed through SDS-PAGE based on the methods of Zhang et al. [28]. The MP solutions (2 mg/mL) were mixed with sample loading buffer (with or without DTT) at a volume ratio of 4:1, boiled at 100 °C for 8 min and cooled. The samples (10 µL) were loaded into the sample wells of a discontinuous SDS-PAGE setup consisting of a 4% acrylamide stacking gel and a 10% separating gel, and then electrophoresed at a voltage of 120 V until the samples arrived at the bottom of the gel. The gel was then stained with 12.5% Coomassie Brilliant Blue R-250 containing 30% carbinol (*v*/*v*) and 10% acetic acid (*v*/*v*) for 2 h and destained with 30% (*v*/*v*) carbinol and 10% (*v*/*v*) acetic acid until the protein bands were clear.

### 2.10. Preparation of Heat-Induced Gel from Bay Scallop Adductor

Fresh and thawed bay scallop adductor muscles were minced via a food processor grinder (Kitchen-Aid Trading Co., Ltd., Shanghai, China) for 3 min, 2.5 g/kg NaCl was added, and the mixture was mixed for 5 min in a cold room (5 °C). The scallop adductor mince was squeezed into plastic casings 25 mm in diameter and 50 mm in length (Shandong Xiongshi Food Co., Ltd., Shandong, China), and both sides were sealed tightly. Scallop adductor heat-induced gels were prepared by preheating the mince in a 40 °C water bath for 30 min and then heating the mince in a 90 °C water bath for 10 min, which has been proven to produce the best gel network in numerous studies [18,29,30]. The prepared gel samples with plastic casings were chilled quickly with ice water and stored at 4 °C overnight prior to analysis.

### 2.11. Textural Properties of Gel from Bay Scallop Adductor

The texture analyses included gel strength analysis and texture profile analysis (TPA), both of which were carried out with a TMS-PRO testing machine (Food Technology Co., Sterling, VA, USA). The gel samples from each group were stripped from the casing and cut into cylinders (25 mm × 25 mm). Each measurement was repeated six times.

The gel strength was determined according to the method of Xie et al. via a 5 mm diameter metal spherical probe with probe dimensions of TA50 [31]. The test program parameters were set as follows: test speed of 60.0 mm/min; trigger force of 10 g; and penetration distance of 10 mm. The gel strength (g·cm) was expressed by multiplying the breaking force (g) and deformation distance (cm).

TPA was conducted according to the methods of Guo et al. with minor modifications [32]. The test was performed with a compression cycle level of 30% at a constant probe speed of 60 mm/min using a cylindrical probe (12.7 mm in diameter and 35 mm long) with probe dimensions of TA10. The TPA parameters, including hardness, springiness, chewiness and reversibility, were obtained.

### 2.12. WHC of Gel from Bay Scallop Adductor

WHC was measured via the method described by Dai et al. [33]. Approximately 3 g of gel was removed, and its weight was recorded accurately as M_1_. The samples were transferred to a centrifuge tube after being wrapped with filter paper. The mixture was then centrifuged at 2685× *g* for 10 min at 4 °C (Hunan Xiangyi Laboratory Instrument Development Co., Changsha, China). After centrifugation, the weight of each sample was recorded as M_2_. The calculation formula is expressed as follows: WHC (%) = M_2_/M_1_ × 100.

### 2.13. Whiteness of Gel from Bay Scallop Adductor

The whiteness of the gels was determined via a CR-400 colorimeter (Konica Minolta, Osaka, Japan). The color parameters, including L* (lightness), a* (redness/greenness) and b* (yellowness/blueness), were measured, and the whiteness values were calculated as follows [34]: whiteness = 100 − [(100-L*) 2 + a*^2^ + b*^2^]^1/2^.

### 2.14. Microstructure of Gel from Bay Scallop Adductor

Sample preparation for microstructure observation was accomplished as previously described [35]. The surimi gels were cut into 5 mm × 5 mm × 1 mm slices and soaked in 2.5% (*v*/*v*) glutaraldehyde at 4 °C for 24 h. Ethanol dehydration was conducted using increasing concentrations of ethanol solutions (50, 70, 90 and three times with 100%) for 15 min per solution. Slices were freeze-dried via a SCIENTZ-12N/A freeze-dryer (Ningbo Scientz Biotechnology Co., Ltd., Ningbo, China), fixed onto a metal stub and sputter-coated with gold. The samples were then scanned via scanning electron microscopy (SEM) (Supra 55, Carl Zeiss, Germany) at an acceleration voltage of 15 kV.

### 2.15. Statistical Analysis

The experiment was repeated at least three times. The data were analyzed via SPSS (SPSS 23.0, IBM, Chicago, IL, USA) and are expressed as the means ± standard deviations (SDs). *p* < 0.05 was considered statistically significant and was established via the Duncan multiple range test. The data were reproduced via Origin 2022 software (Origin Laboratories, Northampton, MA, Inc.), and the correlation coefficients analyzed via Pearson’s two-tailed correlation were plotted to analyze the relationships among the different variables.

## 3. Results and Discussion

### 3.1. Changes in TGase Activity of Bay Scallop Adductor

**Figure 1:**
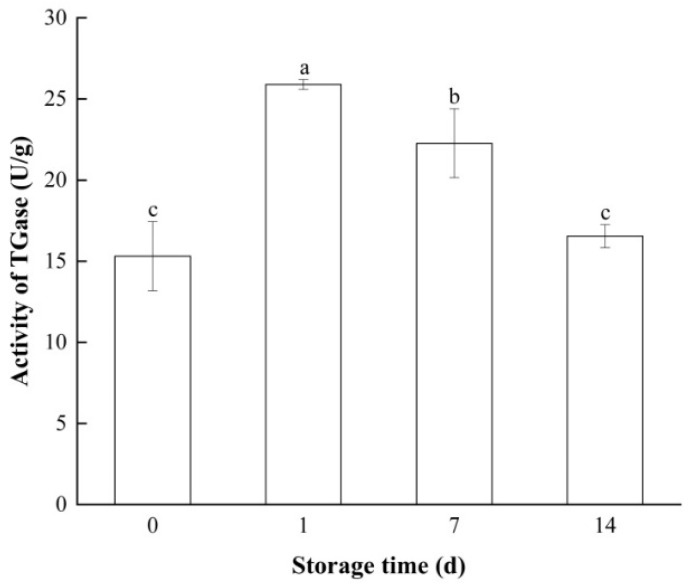
Changes in TGase activity in short-term-frozen bay scallop adductor muscles at −18 °C. Notes: Bars indicate standard deviations from triplicate determinations. Different letters denote statistically significant differences between different storage times (*p* < 0.05).

Endogenous TGase plays an important role in the gelation process [36]. The changes in the TGase activity of bay scallop adductors during frozen storage are shown in Figure 1. With an increasing time of frozen storage, the cells were gradually disrupted, and TGase leaked from the lysosomes into the cell mixture, leading to a significant increase (*p* < 0.05) in activity from 15.31 U/g to 25.89 U/g on the first day. Endogenous TGase catalyzes the cross-linking reaction of MPs during the gel formation stage. Consequently, elevated TGase activity is conducive to enhancing the textural properties and WHC of gels [15]. However, after the 7th day, the activity of TGase gradually decreased, and on the 14th day, the TGase activity decreased to 16.55 U/g, a value close to that of fresh samples. A similar trend was reported by An et al. in their study on endogenous TGase in silver carp (Hypophthalmichthys molitrix) muscle during short-term frozen storage [37]. They reported that the deactivation of TGase led to a significant decline in the textural properties and WHC of the frozen silver carp. It was presumed that the deactivation of endogenous TGase was due to structural changes in the proteins induced by frozen storage [19].

Cysteine and serine proteases have also been identified in bay scallop adductor muscles, and can degrade structural proteins such as MPs and connective tissue proteins to induce modori [38]. The activity of these endogenous proteases increases during the early stage of frozen storage [39]. However, gel softening caused by these endogenous proteases mainly occurs with heating at 50–70 °C [40]. In this study, the roles of proteases in protein degradation were suppressed during two-step (40–90 °C) water bath heating; therefore, endogenous proteases such as cysteine or serine proteases could not play a critical role in degrading the gel. However, the mechanisms by which endogenous proteases affect the structural proteins of short-term-frozen bay scallop adductors remain to be explored in depth.

### 3.2. Changes in Structure and Conformation of Myofibrillar Proteins from Bay Scallop Adductor

#### 3.2.1. Carbonyl Content

**Figure 2:**
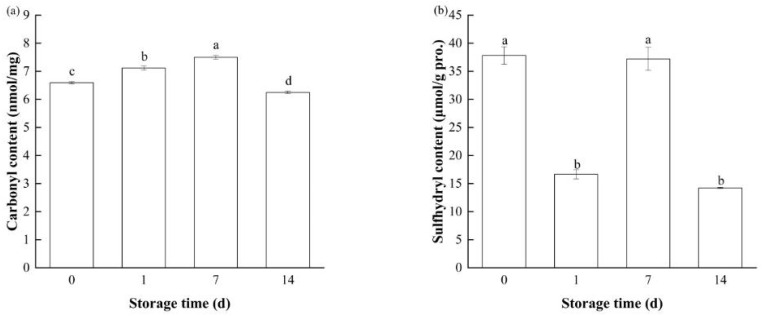
Changes in the (**a**) carbonyl and (**b**) sulfhydryl contents of myofibrillar proteins from short-term-frozen bay scallop adductor muscles at −18 °C. Notes: Bars indicate standard deviations from triplicate determinations. Different letters denote statistically significant differences between different storage times (*p* < 0.05).

Carbonylation represents an important chemical modification of MP oxidation; the carbonyl content is a standard indicator of the degree of protein oxidation [41]. The formation of carbonyl groups in the MPs of bay scallop adductors during frozen storage is shown in Figure 2a. Significant increases (*p* < 0.05) in the carbonyl content from 6.59 nmol/mg to 7.50 nmol/mg were detected in the samples frozen for 7 days, indicating oxidative changes in bay scallop adductors during freezing. The oxidation of protein molecules is caused by certain pre-oxidation factors due to the release of mitochondria, lysosomal enzymes and other pro-oxidation factors into the sarcoplasm with the destruction of bay scallop adductor muscle cells by ice crystals during freezing. The ε-NH_2_ groups from the residues of lysine, proline, arginine and threonine are directly oxidized to form carbonyls by deamination reactions [42]. The formation of carbonyls during frozen storage could lead to the loss of textural, structural and nutritional characteristics of raw materials and, finally, to deterioration in gel properties, rendering surimi-like product unacceptable for human consumption [43]. Meanwhile, although the activity of TGase was still high at day 7, the enzyme-induced cross-linking reaction was significantly affected due to the effects of protein oxidation, which altered the protein conformation, leading to a reduction in the gel properties of samples [19]. When the frozen storage time exceeded 14 days, the carbonyl content decreased to 6.25 nmol/mg, which indicated that protein aggregation caused more wrapping of the carbonyl groups. These findings suggest that changes in protein conformation induced by freezing may be the main reason for the decline in the gel properties of frozen bay scallop adductors after 7 or 14 days of storage.

#### 3.2.2. Sulfhydryl Content

Sulfhydryl groups are key reactive functional groups in MPs that can form disulfide bonds under heat and are important for the formation of protein gels [44]. Additionally, the content of sulfhydryl groups could reflect the degree of oxidation of MPs [45]. The changes in the sulfhydryl content of MPs in bay scallop adductors during frozen storage are shown in Figure 2b. Interestingly, with increasing freezing time, the sulfhydryl content of the MPs first decreased, then increased and then decreased again. Most studies have shown that freezing reduces the sulfhydryl group contents of MPs because of the destruction of the hydrate layers by ice crystals and oxidative reactions. Moreover, the oxidation of sulfhydryl groups contributes to protein cross-linking, leading to deterioration in the gel properties of raw materials during frozen storage [46]. However, changes in the sulfhydryl content of MPs generated by frozen bay scallop adductors during 14 days of frozen storage have not been reported previously. The MP content, activity of TGase, protein structure and protein oxidation level, which are influenced by different raw material species and pretreatment methods, can affect the sulfhydryl content of MPs during frozen storage [37]. Compared with that in fresh samples, the sulfhydryl content in MPs on day 1 of freezing was significantly lower, possibly because the protein molecules were highly hydrated and in a relatively stable folded state.

With increasing frozen storage time, the concentration of sulfhydryl groups in the MPs of bay scallop adductors significantly increased from 16.65 μmol/g protein (day 1) to 37.21 μmol/g protein (day 7), which was strongly associated with the unfolding of protein molecules caused by the growth of ice crystals during freezing [47]. The growth of ice crystals can significantly decrease the degree of hydration of proteins, leading to the unfolding of peptide chains. The sulfhydryl groups in the head and tail of the myosin molecule are subsequently exposed when the conformation of the protein changes [48]. The exposed sulfhydryl groups are more susceptible to oxidation to form disulfide bonds and other oxidized products during frozen storage; therefore, the sulfhydryl content of the scallop samples was significantly reduced to 14.22 μmol/g protein on day 14. The results align with the findings depicted in Figure 2a, which show that the carbonyl group content in MPs indicates an oxidative process occurring during frozen storage. Additionally, the decreased content of sulfhydryl groups on day 14 also could be due to the aggregation of MPs as mentioned above.

#### 3.2.3. Secondary Structures

**Figure 3:**
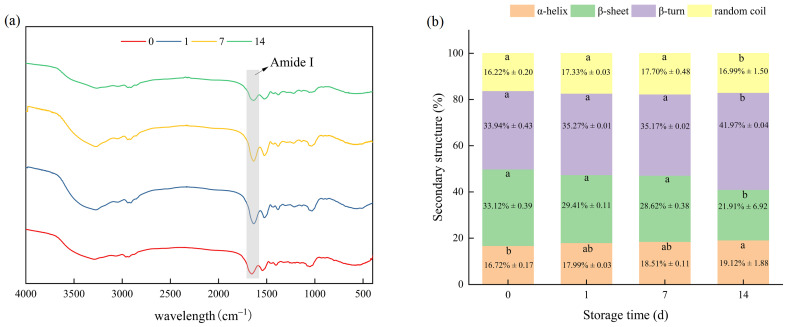
Changes in the FTIR spectra (**a**) and secondary structure (**b**) of myofibrillar proteins from short-term-frozen bay scallop adductor muscles at −18 °C. Notes: Each value is expressed as the mean ± SD (n = 3). Different letters in the same group denote statistically significant differences between different storage times (*p* < 0.05).

FTIR spectra between 4000 and 500 cm^−1^ were used to analyze the changes in the secondary structure of bay scallop adductor myofibrillar proteins during frozen storage, and the resulting spectra are shown in Figure 3a. Changes in the spectral band frequency and intensity indicate significant modifications in the protein molecular structure and local environment of MPs [29]. The secondary structural information of the MPs was derived by fitting the amide I band (1600–1700 cm^−1^) and comparing the peak areas corresponding to α-helices, β-turns, β-sheets and random coils in the proteins. The relative contents of the main secondary structure of MPs from bay scallop adductors are shown in Figure 3b for different freezing times.

No significant differences in protein secondary structure were observed between frozen samples on day 1 or day 7 and fresh samples (*p* > 0.05, Figure 3b). As the freezing time increased to 14 days, the freezing treatment significantly influenced the secondary structure of the MPs. The mass fraction of α-helices increased from 16.72% (fresh sample) to 19.12% (day 14), and the mass fraction of β-turns increased from 33.94% to 41.97%. Moreover, the mass fraction of β-sheets decreased from 33.12% to 21.91%. During frozen storage, the formation and recrystallization of ice crystals may expose the hydrophilic and hydrophobic regions of MPs and affect their refolding and aggregation. Furthermore, with prolonged storage, the disulfide bonds of MPs may form small molecular substances that can weaken the hydrogen bonds in the MPs. Similar changes were reported by Xie et al., who showed that β-sheets continuously decreased and transformed into β-turns in MPs in fish surimi during frozen storage [49]. The low β-sheet content and high α-helix, β-turn and random coil content resulted in greater flexibility in the secondary structure [50]. Folded structures are favorable for exposing intramolecular thiol groups and hydrophobic groups. This exposure subsequently influences the tertiary structure of proteins, resulting in changes in food protein function, which in turn lead to differences in functional properties.

#### 3.2.4. Surface Hydrophobicity

**Figure 4:**
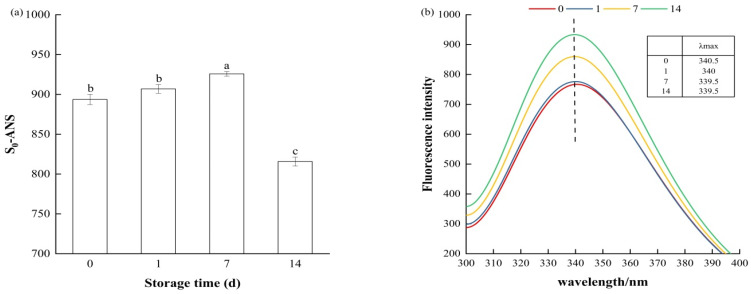
Changes in (**a**) surface hydrophobicity content and (**b**) fluorescence spectra of myofibrillar proteins from short-term-frozen bay scallop adductor muscles at −18 °C. Notes: Bars indicate standard deviations from triplicate determinations. Different letters indicate statistically significant differences between different storage times (*p* < 0.05). 0, 1, 7 and 14: samples frozen for 0, 1, 7 and 14 d, respectively.

Surface hydrophobicity, a measure that reflects the relative content of hydrophobic amino acid residues on the surface of proteins, is often used to assess subtle conformational changes in MPs [46]. Figure 4a shows the surface hydrophobicity of MPs in bay scallop adductor samples during short-term frozen storage, which increased significantly from 893.63 (day 0) to 925.77 (day 7) (*p* < 0.05); this may be due to the unfolding of proteins or breaking of the hydrophobic core, which promotes interactions between hydrophobic groups and the exposure of hydrophobic amino acids, such as tryptophan (Trp) and tyrosine (Tyr), on the surface [51]. The enhancement of hydrophobic interactions makes it more difficult for MPs to combine with water, significantly weakening the gelling strength and WHC of MPs [52]. The growth of ice crystals removes water bound to groups on the surface of the protein, allowing these groups to be released and interact with each other, causing covalent cross-linking and aggregation among protein molecules. Similarly to the carbonyl and sulfhydryl content (Figure 2), the surface hydrophobicity of the samples on day 14 was significantly reduced to 815.73, which may be due to protein aggregation.

#### 3.2.5. Intrinsic Fluorescence Intensity

Aromatic amino acids within proteins inherently emit fluorescence at specific wavelengths. The Trp residue is located in the core of the MPs and has a high FI and maximum fluorescence emission wavelength (λ_max_). Moreover, the position and distribution of Trp in proteins are easily affected by the polarity of the environment. Therefore, FI and λ_max_ can reflect changes in the environmental polarity of Trp residues, which can be used as indicators of changes in the tertiary structure of MPs [9]. As shown in Figure 4b, the λ_max_ of the MPs in the samples decreased from day 0 to day 14 (blueshifted), indicating that the Trp residues were in a hydrophobic environment [43]. The FI of MPs in the samples on the first day did not change significantly compared to the fresh samples. And the FI of MPs increased significantly at day 7 and day 14, indicating an increase in the Trp residue content. The increase in FI values may result from the structural changes in protein molecules and the exposure of hydrophobic microregions caused by the growth of ice crystals during later frozen storage. These results are in line with the analyses of surface hydrophobicity, which indicated that short-term freezing causes changes in the tertiary structure of the MPs in bay scallop adductors. The tertiary structure of proteins determines their physiological activity [53]. Alterations in the tertiary structure of MPs in bay scallop adductor muscles result in changes in their gelling properties.

#### 3.2.6. Sodium Dodecyl Sulfate–Polyacrylamide Gel Electrophoresis

**Figure 5:**
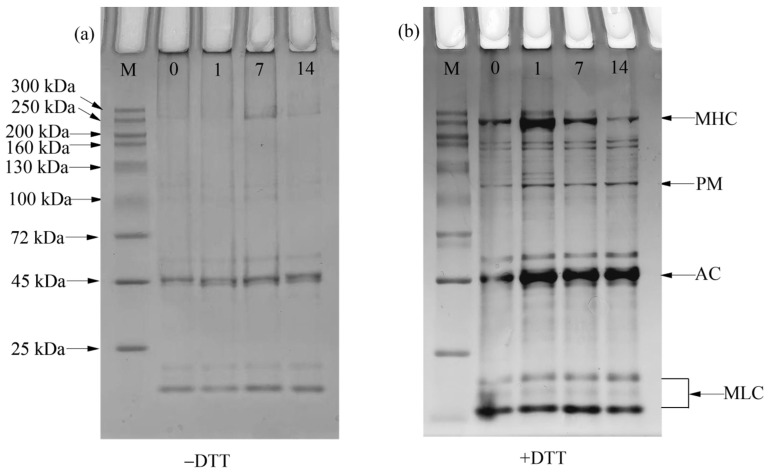
Changes in (**a**) sodium dodecyl sulfate–polyacrylamide gel electrophoresis without dithiothreitol (DTT) and (**b**) with DTT for myofibrillar proteins from bay scallop adductor muscles short-term frozen at −18 °C. Notes: Lane 1—marker; Lane 2–5—samples frozen for 0, 1, 7 and 14 d. MHC—myosin heavy chain; PM—paramyosin; AC—actin; MLC—myosin light chain.

SDS-PAGE is normally used to detect the covalent bonds formed between proteins; changes in electrophoretic bands can reflect protein degradation during frozen storage [47]. The electrophoretic profiles of the MPs from fresh and short-term-frozen bay scallop adductor samples are shown in Figure 5. Myosin heavy chain (MHC, 200 kDa), paramyosin (PM, 116 kDa), actin (AC, 48 kDa) and myosin light chain (MLC, 15–25 kDa) were the four major bands identified via SDS-PAGE. The most important protein related to the gelling properties of the muscle system is MHC [48]. In SDS-PAGE, DTT disrupted the disulfide bond, whereas the samples without DTT produced dark stains at the top of the stacking gels, which is a result of protein oxidation causing the cross-linked aggregation of MPs deposited at the injection port. Most protein bands were not evident in the SDS-PAGE gels without DTT, as shown in Figure 5a, indicating that disulfide bonds were the main cause of protein cross-linking among bay scallop adductor muscle tissues. Disulfide bonds mainly originate from the oxidation of sulfhydryl groups inside protein molecules [44]. Therefore, these results suggest that the sulfhydryl groups in MPs play crucial roles in the formation of protein gels. The reduced MPs were supplemented with DTT, which destroyed the disulfide bonds within the protein. The intensity of the MHC bands of the MPs in the bay scallop adductor samples dramatically decreased and the intensity of the bands with smaller molecular weights increased with an increasing duration of frozen storage, indicating the degradation of MHC.

### 3.3. Changes in the Properties of Bay Scallop Adductor Gel Caused by Frozen Storage

#### 3.3.1. Texture

**Figure 6:**
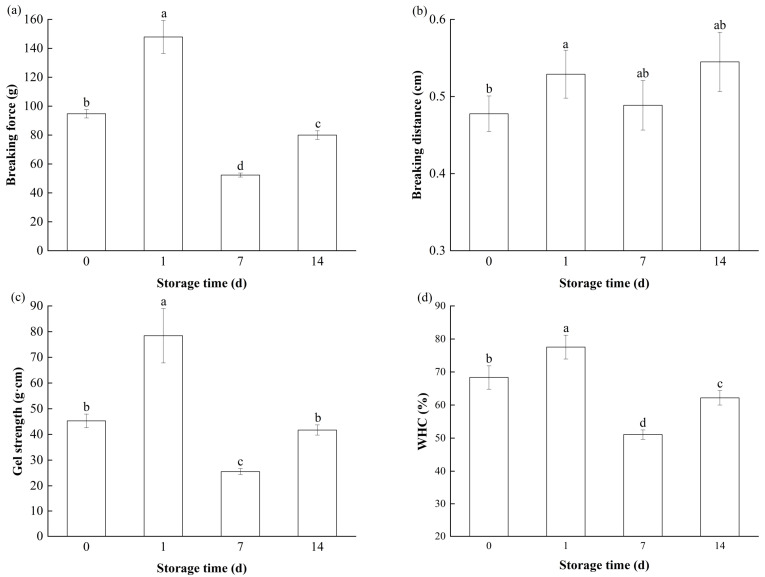
Changes in the (**a**) breaking force, (**b**) breaking distance, (**c**) gel strength and (**d**) water-holding capacity (WHC) of the gels from bay scallop adductor muscles short-term frozen at −18 °C. Notes: Bars indicate standard deviations from triplicate determinations. Different letters denote statistically significant differences between different storage times (*p* < 0.05).

Texture is an important parameter used to evaluate gel quality [54]. The textural properties, including the gel strength and texture profile, of heat-induced gels from fresh bay scallop adductors and those short-term frozen at −18 °C are presented in Figure 6 and Table 1. Similarly to the TGase activity, the gel breaking force, breaking distance, gel strength, hardness, springiness, cohesiveness and chewiness of the fresh sample were 94.73 g, 0.48 cm, 45.25 g·cm, 12.80 N, 0.63, 5.22 mJ and 0.64, respectively, and these values significantly increased (*p* < 0.05) to 147.83 g, 0.53 cm, 78.42 g·cm, 15.08 N, 0.68, 7.06 mJ and 0.68, respectively, on the first day of frozen storage. The increase in gel texture during the first few days was most likely due to endogenous TGase [48]. This finding is corroborated by the study of An et al., who documented an increase in the breaking force and hardness of silver carp (*Hypophthalmichthys molitrix*) surimi gels during the first 5 days of frozen storage [37].

All the texture indicators, except breaking distance, of the gel significantly decreased (*p* < 0.05) on the 7th day. The reduction in the gel texture of bay scallop adductor muscle induced by frozen storage can be attributed to the rupture of the gel network structure. Although the activity of TGase was still high on day 7 (Figure 1), the decreasing gelation texture of bay scallop adductors on the 7th day of storage was evident. Thus, we hypothesized that the change in structural proteins in bay scallop adductor muscle caused by the destruction of hydrate layers by ice crystals and oxidative reactions during frozen storage disrupted the cross-linking of the gel by TGase.

Interestingly, all indicators of the textural properties of bay scallop adductors significantly increased (*p* < 0.05) on the 14th day in comparison with the sample values on the 7th day of frozen storage (Figure 6 and Table 1). Similarly to our results, Lu et al. [55] reported that mild protein oxidation caused by 7 weeks of frozen storage can promote the hardness, chewiness, springiness and gumminess of MP gels from bighead carp. Moderate oxidative modification of MPs could improve the gelling texture by changing the mode of myosin aggregation, which was attributed to a shift in myosin cross-linking at the early stage of heating from head–head association to predominantly tail–tail aggregation through disulfide bonds [14]. According to the series of significant alterations in the oxidation and structure of MPs in bay scallop adductors (Figure 2, Figure 3, Figure 4 and Figure 5), it was presumed that moderate oxidative modification of MPs caused some favorable changes in the gel texture of frozen bay scallop adductors at day 14. However, an enhancement in gelling texture caused by the freezing of bay scallop adductors for 14 days has not been reported previously. The gelling properties of bay scallop adductors during short-term freezing are more prone to changes than those of fish.

#### 3.3.2. WHC

WHC refers to the ability of the scallop gel to retain both inherent and added water [29]. A high WHC is crucial for capturing and retaining sufficient moisture during the cooking process. The WHC of the gels from the bay scallop adductors during frozen storage is displayed in Figure 6d. Similarly to the gel texture (Figure 6c, Table 1), the WHC decreased after an increase, and it reached a maximum of 77.53% during the first day of freezing. The increase in the WHC during early frozen storage is similar to the results for silver carp surimi gel reported by An et al. [37]. The enhanced gel strength enables the gel’s network structure to retain a greater quantity of water, thereby facilitating stronger gel cohesion. Compared with that of the fresh sample, the gel of the sample on the first day had a better gel texture (Figure 6c, Table 1), leading to an increase in the WHC.

The oxidation of MPs caused by frozen storage promotes the aggregation of myosin and actomyosin, which can assemble into an irregular and heterogeneous gel and decrease the WHC [56,57,58]. However, in this study, the WHC of the gel samples on the 14th day was 62.18% lower than that of the fresh samples, which was significantly greater (*p* < 0.05) than that of the samples on the 7th day. Numerous studies have demonstrated that mild protein oxidation can increase the WHC of MPs [14,55]. The data in previous subsections (Figure 2, Figure 3, Figure 4 and Figure 5) have confirmed that significant protein oxidation and structural changes occurred in the MPs of the bay scallop adductor muscle on the 14th day. Therefore, the increase in WHC on the 14th day could be a consequence of protein–protein and protein–water interaction caused by moderate oxidative modifications to the MPs of frozen bay scallop adductor muscles during frozen storage.

#### 3.3.3. Whiteness

Whiteness is an important quality index of gels [59]. As shown in Table 1, the whiteness of the gels from the bay scallop adductor on day 1 significantly increased (*p* < 0.05) compared with that of the fresh samples. TGase facilitates the formation of cross-links between protein molecules, thereby increasing the whiteness of protein gels [18]. In general, the process of ice crystal formation during the freezing of samples leads to the disruption of muscle cell membranes, which contributes to the degradation of proteins and a reduction in gel whiteness [30]. However, the whiteness of the gels from the bay scallop adductors clearly increased with increasing frozen storage time, with the whiteness reaching a maximum of 73.41 on the 7th day. Notably, there was no significant difference in whiteness between the gels on the 7th and 14th days. This phenomenon may be attributed to the fact that the generation of ice crystals, changes in free water and the reflection of light on the gels have a more pronounced impact on whiteness than changes in the structure of MPs [60].

### 3.4. Changes in Microstructure of Gels from Bay Scallop Adductor

**Figure 7:**
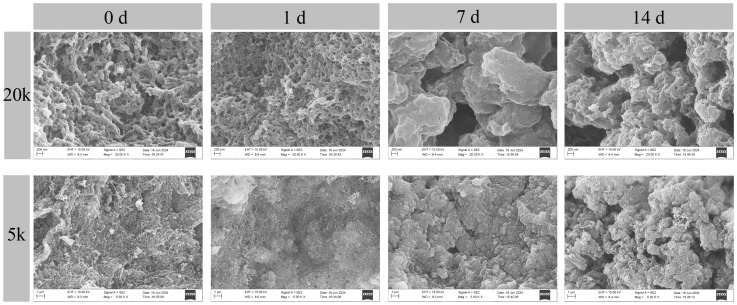
Changes in the microstructure of gels from bay scallop adductor muscles short-term frozen at −18 °C.

The texture and WHC of a gel depend on its microstructure to a certain degree, particularly the homogeneity and compactness of the gel network [14]. The changes in the microstructure of the gels from the bay scallop adductor are shown in Figure 7. A regular dense structure with a well-organized three-dimensional network was observed in the gel from the fresh sample. TGase promotes the cross-linking of MPs and produces a firmer and more stable gel structure [19]. Consistent with this report, the higher TGase activity observed in frozen bay scallop adductors on the first day (Figure 1) led to the formation of a more compact and porous gel network. This microstructural change resulted in the superior textural properties and increased WHC of frozen bay scallop adductors on the first day (Figure 6 and Table 1). On the 7th day, the samples had a nonuniform network with voids and cavities, suggesting that frozen storage resulted in the poor gel-forming ability of the bay scallop proteins, which was also consistent with the gel texture properties and WHC results. These observations are likely due to the partial aggregation of unfolded MPs induced by oxidation during frozen storage [61]. Li et al. reported that moderate protein oxidation causes the formation of covalently cross-linked structures of MPs, reducing the number of pores and changing the structure of the aggregate gel, eventually resulting in the formation of a smaller aggregate gel network [10]. This may also explain why the microstructure of the gel on the 14th day was better than that on the 7th day.

### 3.5. Correlation of TGase Activity, Protein Structure and Gel Properties of Frozen Bay Scallop Adductor

**Figure 8:**
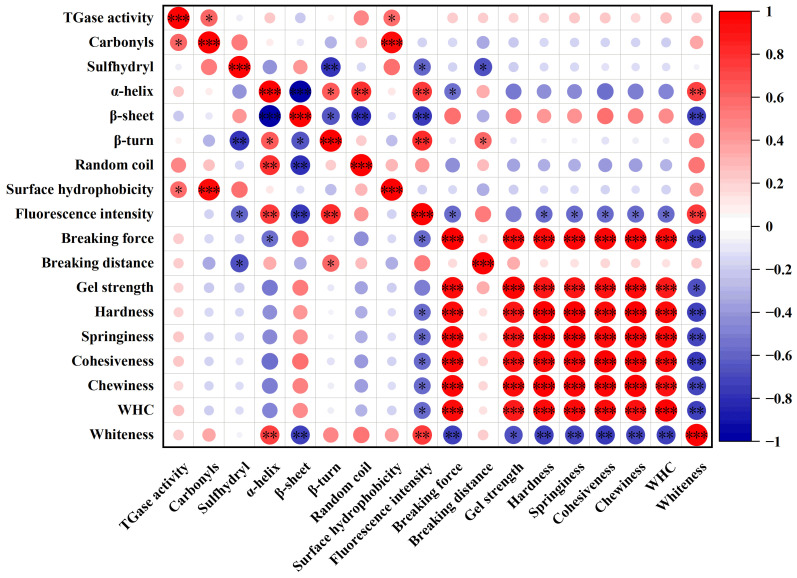
Heatmap of correlations among transglutaminase (TGase) activity, the structure of myofibrillar proteins and the properties of heat-induced gel from bay scallop adductor muscles short-term frozen at −18 °C. Notes: *** indicates a significant correlation at the 0.001 level; ** indicates a significant correlation at the 0.01 level; * indicates a significant correlation at the 0.05 level; red represents positive correlations; blue represents negative correlations. The intensity of the color reflects the strength of the correlation, with darker shades signifying stronger relationships.

To investigate the associations among endogenous TGase, the MP structure and the gel properties of frozen bay scallop adductor muscles, a correlation analysis of the detected indices was conducted. The results are shown in Figure 8. TGase activity was significantly positively correlated with the carbonyl content and surface hydrophobicity of MPs (*p* < 0.05), indicating that the deactivation of endogenous TGase was likely a result of protein structural modifications caused by frozen storage. In the heat-induced gelation process at 40 °C, the MPs were polymerized via TGase catalysis, resulting in a dense and homogeneous three-dimensional network structure and the formation of a good gel texture. Therefore, the activity of endogenous TGase could affect gel quality [47]. However, TGase activity was not significantly positively correlated with any of the gel properties, such as breaking force, gel strength, hardness, springiness, cohesiveness, chewiness or whiteness, of the heat-induced gel produced from the frozen bay scallop adductor (*p* > 0.05). This phenomenon can be attributed to the fact that TGase catalyzes protein cross-linking, a process that is highly dependent on the structure of MPs. This dependency is further illustrated by the observation that on the 7th day of storage, despite high TGase activity (Figure 1), the gel texture was notably poor (Figure 6 and Table 1).

As a common indicator of protein oxidation, the sulfhydryl content was significantly negatively correlated with the contents of β-turns (*p* < 0.01) and FI (*p* < 0.05), indicating that the sulfhydryl content is functionally important for stabilizing the spatial structures of MPs. Moreover, the sulfhydryl content was significantly negatively correlated with the breaking force of the heat-induced gel (*p* < 0.05), whereas the carbonyl content was not significantly correlated with any indicator of gel properties (*p* > 0.05), indicating that the sulfhydryl group is the key to influencing the gel indicators, which is also consistent with the SDS-PAGE results (Figure 5).

The α-helix content was significantly negatively correlated with the gel breaking force (*p* < 0.05) and positively correlated with gel whiteness (*p* < 0.01). The β-turn content was significantly positively correlated with the gel breaking distance (*p* < 0.05). The analysis also revealed that the indicator of protein tertiary structure, FI, was significantly negatively correlated with the gel breaking force, texture profile and WHC (*p* < 0.05) and significantly positively correlated with gel whiteness (*p* < 0.01). FI significantly increased at days 7 and 14, indicating structural changes in the protein molecules (Figure 4b). Some studies have shown that changes in protein structure during frozen storage could result in textural loss in aquatic materials [43]. The correlation between the MP structure and gelling properties of frozen bay scallop adductor muscles also demonstrated that the structural changes in MPs of bay scallop adductor muscles induced by frozen storage directly affect their gelling properties.

## 4. Conclusions

During the short-term freezing process, the activity of endogenous TGase and the structure of MPs significantly influenced the gel strength, texture properties, WHC and microstructure of heat-induced gel from bay scallop adductors. Under the influence of endogenous TGase, the gel of the moderately frozen bay scallop adductor (1 day) exhibited significantly better properties than that of the fresh bay scallop adductor, with significantly better breaking force, gel strength, hardness, springiness, cohesiveness, chewiness and WHC, as well as a more compact gel microstructural network. In addition, during frozen storage, MPs underwent oxidative denaturation and their structure changed significantly at day 7 and day 14. Meantime, the TGase activity of bay scallop adductors decreased, and the cross-linking effect on the protein was also affected by changes in the protein molecular structure. These changes collectively led to significant deterioration in the gel texture, WHC and microstructure of bay scallop adductors. This study provides new ideas for the processing of frozen bay scallop adductors and the production of frozen surimi-like products.

## Figures and Tables

**Table 1:** Changes in the texture and whiteness of the gels from bay scallop adductor muscles short-term frozen at −18 °C.

Freezing Time (d)	Hardness (N)	Springiness	Chewiness (mJ)	Cohesiveness	Whiteness
0	12.80 ± 0.70 ^b^	0.63 ± 0.01 ^b^	5.22 ± 0.33 ^b^	0.64 ± 0.01 ^b^	69.53 ± 0.41 ^c^
1	15.08 ± 0.75 ^a^	0.68 ± 0.01 ^a^	7.06 ± 0.56 ^a^	0.68 ± 0.01 ^a^	71.40 ± 0.26 ^b^
7	8.10 ± 0.17 ^d^	0.42 ± 0.02 ^d^	1.60 ± 0.18 ^d^	0.47 ± 0.02 ^d^	73.41 ± 0.15 ^a^
14	10.7 ± 0.45 ^c^	0.54 ± 0.02 ^c^	3.29 ± 0.27 ^c^	0.57 ± 0.02 ^c^	73.25 ± 0.01 ^a^

Notes: Each value is expressed as the mean ± SD (n = 6 for texture or 3 for whiteness analysis). Different superscript letters in the same group denote statistically significant differences between different storage times (*p* < 0.05).

## Data Availability

The original contributions presented in the study are included in the article, further inquiries can be directed to the corresponding author.
